# Child health, household environment, temperature and rainfall anomalies in Honduras: a socio-climate data linked analysis

**DOI:** 10.1186/s12940-020-0560-9

**Published:** 2020-01-28

**Authors:** Cristina Bradatan, Jeffrey A. Dennis, Nadia Flores-Yeffal, Sharmistha Swain

**Affiliations:** 1 Department of SASW, Texas Tech University, Holden Hall 158, Lubbock, TX 79409 USA; 2 Climate Science Center, Texas Tech University, Lubbock, USA; 3 Department of Public Health, Texas Tech University Health Sciences Center, Odessa, USA

**Keywords:** Climate anomaly, Child health, Central America, Honduras

## Abstract

**Background:**

As climate research continues to highlight the global shifts in temperature and precipitation, more research is needed to understand how climate anomalies impact human health outcomes. In this paper, we analyze one of the paths through which climate anomalies affect health (in particular, child’s health) within one of poorest countries in the world (Honduras).

**Methods:**

Using the GPS location of the household, we link information on child health and house amenities from the Honduras Demographic Health Survey 2011–2012 dataset (a nationally representative sample) with climate data (1981–2012) from the Climate Research Unit (CRU TS3.21). We use generalized estimating equations for binary logistic models and spatial association to analyze these data.

**Results:**

We show that 1) areas experiencing significant temperature anomalies are also the ones with the worst child respiratory problems and 2) in households with poor amenities – such as access to sanitation and clean water, children tend to have a high incidence of respiratory diseases and diarrhea .

**Conclusions:**

We conclude that, as climate change increases the incidence of climate anomalies, tackling in advance those household environmental factors responsible for poor child health outcomes (better sanitation and clean cooking fuel) can prevent a further deterioration of children’s health in Honduras.

## Background

There are two types of effects climate change is expected to have on human health: 1) direct impacts, due primarily increasing frequency of extreme weather such as heat, drought, and heavy precipitation; 2) mediated effects (such as air pollution, water-borne illnesses, and malnutrition) [1].

While all populations may potentially face direct health effects of climate change, the impacts mediated by natural and human systems are expected to be more substantial for vulnerable populations (children, elderly, poor, indigenous) because of their living conditions. A large percentage of developing countries’ populations, for example, work and subsist on a diet based in non-irrigated agriculture and, as a result, climate changes will negatively impact their economic capacity (i.e., ability to work) and significantly reduce their access to nutritious food, cooking fuel, and clean water [2]. Many of these poor countries suffer from endemic corruption and lack a welfare support, such as unemployment benefits and food stamps [3]. The cumulative impact of these risks makes those more vulnerable to infectious diseases [4, 5] and, as a result, reduces their working capacities and increases expenses, pushing them further into poverty. Extreme poverty has a significant effect as well on the household environment in which these populations live through the materials they use to build their homes and lack of proper access to utilities (such as gas, clean water, and electricity). Therefore, the household environment can also have a negative effect on the health of those individuals living in those homes.

In this paper, we use the 2011–12 Honduras Demographic and Health Survey (DHS) and Global Positioning System (GPS) linked climate data to analyze the relation between child health, household environment and climate anomalies in one of the poorest Central American countries, Honduras. We focus on Honduras for several reasons: 1) it is one of the least developed/poorest countries in Latin America; 2) it has significant environmental issues (deforestation, flooding); 3) children under 15 represent a high percentage of its population (32.95% in 2014, CIA, The World Factbook); 4) almost 40% of population is employed in agriculture (CIA, The World Factbook); and 5) there are a limited number of analyses focused on this country.

In the following pages, we first discuss what we know so far about the impacts of climatic variations on health. Secondly, we connect household health, basic sanitation and water source survey data with climate data to analyze the relationships between environment, household characteristics, and child health in Honduras. Third, we discuss what these results mean for the Honduran children’s health outcomes and what policy recommendations can be made based on our analyses.

Honduras has one of the youngest and fastest growing populations in Latin America (1.6% rate of growth in 2017, 32.95% under the age of 14) with an annual rate of urbanization of 3% (CIA World Factbook). Local governments have limited resources and are often corrupted, so they are unable to keep up with this population growth to offer the services people needed (running water, sewage, waste collection, security) [3]. High levels of violence, large storms, hurricanes (Mitch in 1998), and flooding (NUD, 2011) are factors that have deteriorated the living environment and prevented further the people in Honduras from having access to the health services they need.

Climate change is likely to increase the impact of existing environmental problems worldwide, and Honduras has already experienced significant weather related losses. The damages inflicted by hurricane Mitch led Germanwatch to list Hondurast among the top three countries that suffered the greatest damages during 1992–2011 due to extreme weather events [6]. Due to its geographical position, Honduras is exposed to tropical storms/ hurricanes, floods and landslides. This climate vulnerability is likely to increase in the future as mid-latitude regions such as Honduras are expected to see more extreme precipitation events in the next 80–90 years. These extremes may vary between droughts and flooding at different times [7]. The mean temperature (which is already above 75 °F, while it reaches 93 °F in some areas in Honduras) is expected to increase by 1.0 to 2.5 °C in Central America by 2050 under *all* IPCC emission models [7].

Climate change will also lead to a higher probability of significant flood events [3]. For example, a study of the Choluteca Department (Honduras) found that during 1988–2013, the local population experienced moderate to extreme vulnerability due to natural weather related disasters such as extreme flooding, mud slides, and drought [8]. Both drought and flood episodes have occurred repeatedly during 2000–2011; the 2010 wet season was among the wettest but it was also too short, leading to an abrupt flood-to-drought transition [3]. For the western part of Honduras, however, climate change is expected to lead to a 10–20% decrease in precipitation by 2050, posing significant problems to the local agriculture, especially around the “Corredor seco” (“Dry corridor”) - an area that includes departments of Choluteca, Morazan and Valle [9].

Climate change is expected to have a significant effect on agriculture, the ability of people to work, quantity and quality of food available and, in turn, will affect people’s health especially in countries where large proportions of people (almost 40% in Honduras) are involved in agriculture ([10–13] 2011 [14];). In 2003, when Europe recorded some of the hottest summers in modern history, at least 22,000 deaths were attributed to extreme temperatures. A U.S. study found that negative temperature extremes increased mortality in the warmer (southern) states, whereas in the colder (northern) states, mortality was more affected by the increase in high temperatures [15]. Research done in Peru shows that significant increase in the ambient temperature (brought by ENSO) affects the prevalence of diarrhea among children [16]. On a global level, future climate change may increase diarrhea incidence by up to 29% [13]. Increase in the sea surface temperature has been also shown to be correlated to an increase in the number of cholera cases in the Bay of Bengal [17]. In Honduras, climate change has contributed to a higher prevalence of respiratory diseases, malaria, dengue, and diarrhea. The health of children (0–4) and adults (60+), particularly the poor ones, is more likely to be impacted by these changes [18].

Increasing urbanization, on the other hand, leads to an even higher temperature increase than the one brought by climate change itself. Changes in land cover (from vegetation to asphalt roads and buildings) bring up urban “heat islands,” with temperatures 5–11 °C higher than the surrounding rural areas [19, 20]. In this respect in Honduras, during 1990 to 2005, forest land cover decreased by 37.1%, (the fourth-largest percentage loss for any nation) [3]. Tegucigalpa, the largest city in Honduras, has quadrupled in size since the 1970s and it has a high population density (14,500 people per sq. mile in 2014 210 per sq. mile mean density in Central America ). Population density is especially high in the urban periphery, a part that is also constantly being impacted by floods, landslides and droughts. Many of the poor live in these unplanned settlements, making them especially vulnerable to environmental effects (Adaptation Fund 2015). As more people move to urban areas [21], a continuation of unplanned urban expansion will result in new developments in areas with high environmental risks [22].

Climate changes will also negatively impact already existing environmental problems. There is a significant international literature linking child incidence of diarrhea and cough to unfavorable household environment related to water source, wastewater sanitation and cooking fuel type, respectively [23, 24]. Poor air quality and lack of access to clean water and sanitation increase the prevalence of respiratory diseases, diarrhea, and other infectious diseases (such as dengue and malaria) (PNUD and [18]). In Honduras, for example, in the summer of 2013, the high incidence of dengue fever made the Honduran authorities declare an emergency situation [25].

The type of cooking fuel used in the household proves to be an important factor affecting the incidence of respiratory diseases. Research done in Lucknow (India) showed that the use of dung cakes as cooking fuel has had a significant effect on the incidence of respiratory diseases among children [26].

Similarly, children who don’t have permanent access to clean water and live in generally poor sanitary conditions are more exposed to bacteria and, as a result, have a higher probability of developing infectious diseases. Fink et al. [23] show that access to clean water and sanitation is associated with a lower risk of child diarrhea and a lower risk of mild or severe stunting. Suboptimal nutrition and infectious diseases, in turn, are factors that significantly affect the weight and height of children older than 6 months regardless of the children ethnic and racial background [27].

Climate change, and anomalies in temperature and rainfall in particular, affect directly and indirectly human health. Directly, high heat (above 100/104 F) puts pressure on the human body, it brings heat exhaustion and physical and mental health issues and can lead to death, especially when the person has a pre-existing condition ([28, 29]. Indirectly, climate influences the rate of growth, transmission, or virulence of large pathogens [1]. Lack of access to sanitation and clean water exposes humans to these climate-sensitive pathogens.

Several studies show that respiratory diseases and diarrhea are correlated with variables such as temperature and humidity extremes ([30, 31]; Chang et al., 2012 [32, 33];). This type of research is either done at a macro level or, when done at a micro level, studies are based on hospital admissions that don’t link the place where the child lived before being admitted to the hospital with the meteorological occurrences at his/her place of residence.

## Methods

In this study, we investigate the links between precipitation and temperature values and anomalies, household environment and child health outcomes using a nationally representative sample of Hondurans. Our analyses aim to understand how the context in which individuals live (household features) and climate characteristics affect child health outcomes. We focus here on households with young children (age under 5), who may be most vulnerable to health problems associated with poor access to clean water, sanitation, the intensity of cooking smoke in their home dwelling and temperature and precipitation characteristics of their area of living. We focus on three specific child health outcomes –cough, diarrhea and infant mortality.

### Hypotheses

The literature reviewed above suggests that access to clean water, clean air and sanitation have a significant influence on child’s health, in particular for diarrhea and cough prevalence. We expect that changes in the climate/environment such as increase in precipitation also influence the incidence of infectious and respiratory diseases. Therefore, in this paper we will be testing for the following hypotheses:

H_1:_ Young children living in households that use wood fuel for cooking in the home have higher rates of cough or respiratory illness than those who do not use wood fuel.

H_2:_ Young children living in households with poorer living conditions (such as lack of sanitation [i.e., access to clean water and sewage] and dirt floors) have higher rates of child diarrhea than those who have better living conditions.

H_3:_ Families living in households with harsher household environment (dirt floor, no flush toilets, limited access to clean water, use wood for fuel) experience higher levels of infant mortality.

H_4:_ Areas with greater precipitation and temperature anomalies have poorer child health outcomes.

With these hypotheses in mind, we first explore levels of access to basic needs drinking/current water, sanitation/toilet facilities, type of flooring in the home (such as dirt floor), and the use of firewood for cooking in the home. Secondly, we link climate data (precipitation and temperature) to household and health information using the GPS location of the household.

### Household data

**Fig. 1:**
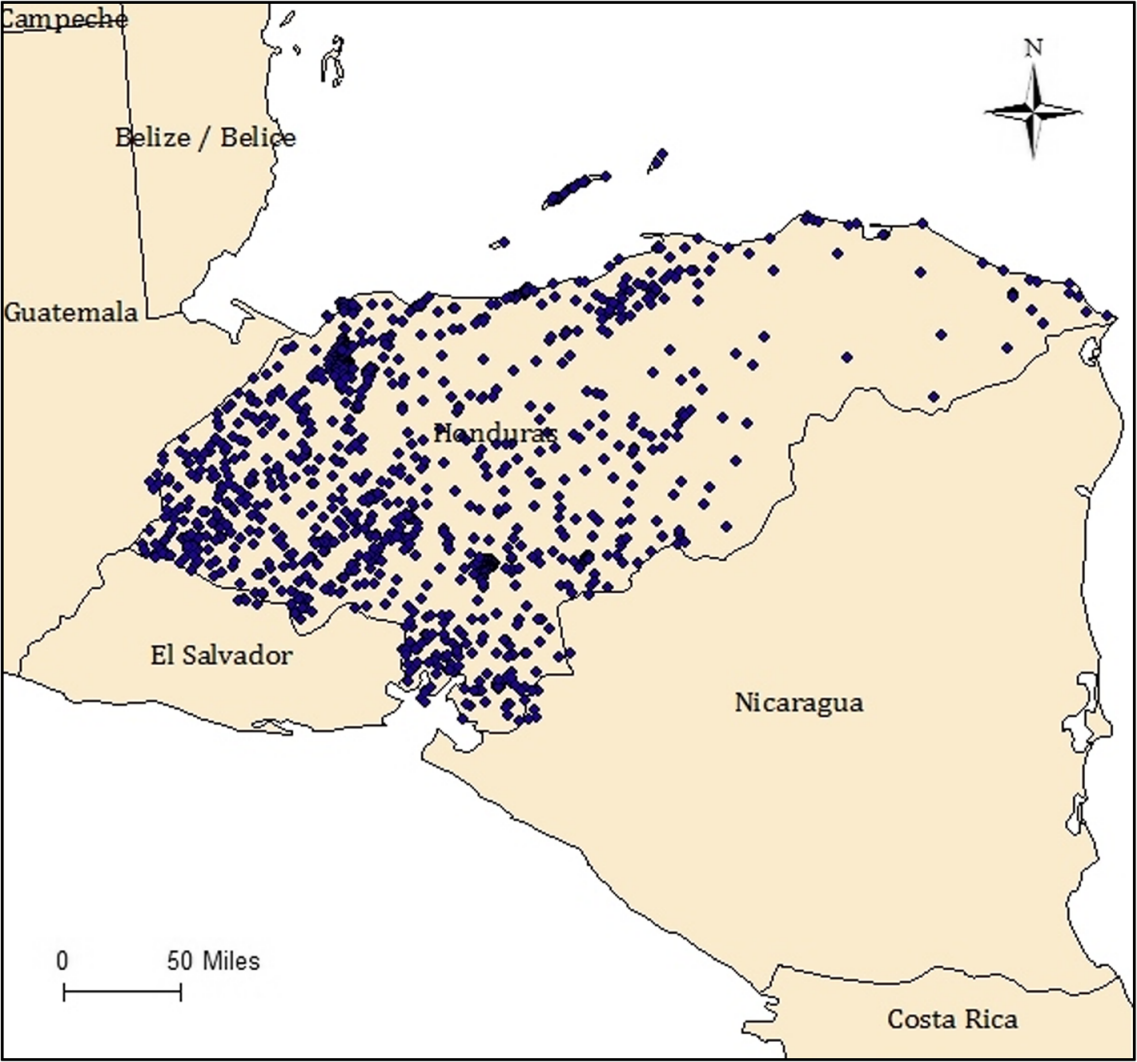
Geographical distribution of Honduran households as surveyed by DHS 2011–12

For data, we use Honduras DHS 2011–2012 dataset that includes the GPS location of the surveyed household (Fig. 1). The 2011–2012 Honduras DHS includes information on about 21,300 households whose residents were interviewed over a two-year period, with specific survey modules for household characteristics as well as demographic characteristics for individual household adults and children. In particular, detailed health data was collected for children under 5 years of age. Sample weighting is used with appropriate survey modules to account for complex survey design.

The GPS household information allowed us to connect child health outcomes and household environmental issues to precipitation and temperature anomalies from the gridded climate data where the households are located (for the precise date when the respondents were interviewed). We use indicators that included climate data for the period of time immediately before the interview relative to long term climate data to analyze how climate deviations (or anomalies) may impact children’s health.

Of the 21,362 households in the survey, 8430 contain information on at least one child under 5 living in it. In order to measure our variables of interest, we use composite child health indicators, indicating whether any child under 5 who lives in the household: 1) has had diarrhea in the past 2 weeks (of the day of the survey) or 2) has had a cough in the past 2 weeks, or 3) whether there is any recorded case of infant mortality (from woman respondent’s births history).

### Climate data

High density and comprehensive observational networks of meteorological data or high spatial resolution gridded data would have facilitated our analyses, but these data are not available for Honduras. Therefore, in this study we used relatively coarse resolution gridded (0.5° by 0.5° latitude and longitude; ~ 53 kms) monthly total precipitation, average temperature, and maximum temperature data for 1981–2012 period from the Climate Research Unit^1^ (CRU TS3.21; Harris, et al., 2014).We need the whole 30 years of data in order to detect climate anomalies. The gridded dataset is generated from monthly observations at meteorological stations covering the global land surface. CRU data have been widely used in a variety of climate and public health related studies (e.g., [34–37]). For this analysis, we performed a bilinear interpolation on the original data (0.5° by 0.5° grids) to develop a re-gridded CRU database at a resolution of 0.025° by 0.025° latitude and longitude (~ 3-km).

### Statistical analysis

In order to analyze the connection between child health and household environment (the first three hypotheses), we start with a chi square analysis. Then we use generalized estimating equations for binary logistic regression models predicting the odds ratios for the three binary measures of child health (cough, diarrhea and infant mortality). The DHS 2011–12 nationally representative sample is a clustered one: it used the 2001 Honduran census framework to divide the population into sectors (based on census tracts) and then select a representative bi-stratified sample of households from within these areas. There are 1119 clusters with 1–26 households where there is at least one child under age 5. We use generalized estimating equations method to take into account the problem brought by the possible lack of independence within the data (Ziegler, 2011). The use of GEE is adequate to our analyses because: 1) households located within the same cluster are more similar to each other than to the others (for more details on the dataset structure, please see the data description above); 2) the number of clusters is large (1119) and 3) we are interested in average population rather than individual outcomes (Ziegler, 2011 [38];).

Household characteristics variables were built using multiple DHS variables. Household cooking fuel was coded to identify any household using wood for cooking fuel, and those households were divided into three groups: 1) those cooking with wood outdoors, 2) those cooking with wood indoors with a chimney, and 3) those cooking indoors with no chimney and then leaving not using wood for cooking as a reference category. Water source was coded to indicate households with water piped into the home (used as reference category), those who obtain water from a pipe somewhere outside the home, from a well, from a stream, lake, or river, or those who use bottled water. Household flooring type was coded as having a dirt floor, a mud or plank floor, or a tile or granite floor (used as a reference category). Toilet type in the household was coded as having a flush toilet (used as a reference category), a latrine, or no toilet at all. Child insurance status was included as a control (Insurance Yes = 1; else =0) to examine health care access, although its prevalence was low (< 10%) for the sample. Mother’s education (first woman listed in the household) has been used as a control variable and it is highlighted when significant in the models. The database has a built-in Wealth index variable (using several household amenities features) but no income variable, so we only used education for a measure of socioeconomic status, as none (reference category), primary, secondary and higher. Urban (versus rural) residence (in which Urban = 1; Rural = 0) is important and can influence child health in two opposite directions: on one hand, urban residences tend to have better household amenities (access to clean water, sewage); on the other hand, urban households might be more exposed to extreme climatic events (e.g. heat islands).

The fourth hypothesis is the one for which we needed both child health and climate data. In order to perform a more reliable statistical analysis, we had to take into account the structures of both the climate and health datasets.

In terms of climate related data, we used climate indicators (such as anomalies in monthly precipitation, maximum temperature, and average temperature) as well as weather characteristics (precipitation and temperature during the month preceding the interview) and analyzed their impacts on children’s health. We calculated climate anomalies (corresponding to the month of survey data collection) using the CRU data. We then overlaid the GPS points on the climate indicator maps and extracted the grid cell values corresponding to the GPS points. So only the GPS points (within same month of data collection) that fall on the same 3-km grid would have the same climate data. Precipitation anomalies were calculated as the current month’s total precipitation minus the average total precipitation for that month for 1981–2010 period, divided by the current month’s total precipitation. Temperature anomalies were calculated as current month’s maximum and average temperatures minus their respective 1981–2010 monthly averages. We then calculated the climate indicators at the cluster level and performed spatial correlation analyses between the child health outcomes and climate data using the SpatialPack package in R [39].

We are aware that, within the social science literature, it has become customary to use multilevel models when climate and social data are combined. However, we agree with Hubbard et al. [38] that the multilevel approach, at least for the type of data used in this research, involves some unverifiable and even biased assumptions so we used generalized equations models.

## Results

Table 1 presents prevalence of child health outcomes by household sanitation, water, and structural characteristics for the whole Honduran sample. Clear disparities are present for households where a child had a cough or diarrhea in the past 2 weeks, providing preliminary support for hypotheses 1 and 2. Households with water piped into the home and those using bottled water have the lowest rates of child cough and diarrhea, with higher rates among those obtaining water from an outdoor pipe, well, or river/lake.

**Table 1:** Weighted percentages by household chracteristics and child health outcomes

	Any child in HH w/ cough	Any child in HH w/ diarrhea	Any infant mortality in HH
Water			
Piped into home	39.69	18.11	2.58
Piped outside of home	44.33	23.12	3.62
Well	49.76	21.45	3.44
Stream/Lake/River	44.08	23.57	3.38
Bottled water	33.77	17.31	2.5
Chi square	82.860	39.020	7.428
p value	0.0001	0.0001	0.153
Flooring			
Dirt floor	47.68	23.79	3.85
Mud/plank floor	40.81	20.9	3.1
Tile/granite floor	33.21	17.34	2.23
Chi square	89.394	26.381	9.330
p value	0.0001	0.0001	0.0177
Cooking fuel			
Not wood fuel for cooking	34.44	17.63	2.5
Wood fuel outside home	44.99	23.19	2.91
Wood fuel inside w/ chimney	42.96	22.18	3.2
Wood fuel inside w/o chimney	46.94	22.69	4.71
Chi square	85.709	30.865	11.892
p value	0.0001	0.0001	0.019
Toilet			
Flush toilet	36.29	17.56	2.91
Latrine	43.45	22.87	2.8
No toilet	47.89	26.53	4.18
Chi square	64.969	55.249	5.481
p value	0.0001	0.0001	0.0924

Households with dirt flooring, as expected, have the least favorable health outcomes, with the highest prevalence of child cough, diarrhea, and infant mortality (47.68, 23.79, and 3.85) respectively. Households using wood fuel for cooking also have unfavorable health outcomes (44.99, 42.96, 46.94) compared to those not using wood fuel (34.44), although the differences between the weighted percentages of those using wood inside or outside the home are not substantial. This suggests that wood cooking fuel may be a proxy for other resources not measured here. Hypothesis 3 is partially supported, as flooring type and cooking fuel are associated with infant mortality, although water source and toilet type do not show a significant association. Burning of firewood is the preferred method used for cooking and heating in Honduras, as 90% of rural and 50% of urban households use it on a daily basis (analysis not shown). Use of firewood leads to high levels of polluted indoor air which, in turn, may increase the incidence of respiratory diseases [40].

We used the generalized estimating equations (GEE) method for binary logistic models to predict odds ratios for the three binary dependent variables (results shown in Tables 2, 3 and 4). Model 1 in Tables 2, 3 and 4 is the basic model showing the relationship between the dependent variable of interest and household conditions, with stepwise additions of urban location, flooring type, and maternal education or insurance coverage, in Models 2, 3 and 4 respectively. Adult education and a composite household wealth index were examined separately in the models, and then together. Household wealth was highly correlated with a number of the household characteristics, and ultimately was excluded due to a lack of significance. Wealth index was a significant predictor of the three dependent variables at the bivariate level, but the variables indicating cooking fuel, wastewater sanitation, water source, and flooring type seemed to cancel out any effects of wealth index, so it has been excluded from the models. Maternal education and child insurance coverage were included in Model 4 for all three dependent variables, although when not statistically significant, they were dropped and the model was re-rerun without them. Ultimately, maternal education was included only for the model predicting a child with diarrhea (Table 3), and insurance was included only for the model predicting child coughs (Table 2).

**Table 2:** GEE logistic regression odds ratios predicting any child in the household with a cough

	Model 1	Model 2	Model 3	Model 4
Cooking fuel (not wood=ref)				
Wood fuel outside home	1.51***	1.46***	1.34***	1.30***
Wood fuel inside w/ chimney	1.40***	1.37***	1.23***	1.22*
Wood fuel inside w/o chimney	1.73***	1.58***	1.45***	1.40**
Urban		0.96	1.02	1.08
Dirt floor (ref=tile/granite floor)			1.49***	1.47***
Mud/plank floor			1.28***	1.26***
Child covered by insurance				0.75
Constant	0.52***	0.53***	0.44***	0.46***

*N* = 8,107

*p < 0.05, **p < 0.01, ***p < 0.001

**Table 3:**
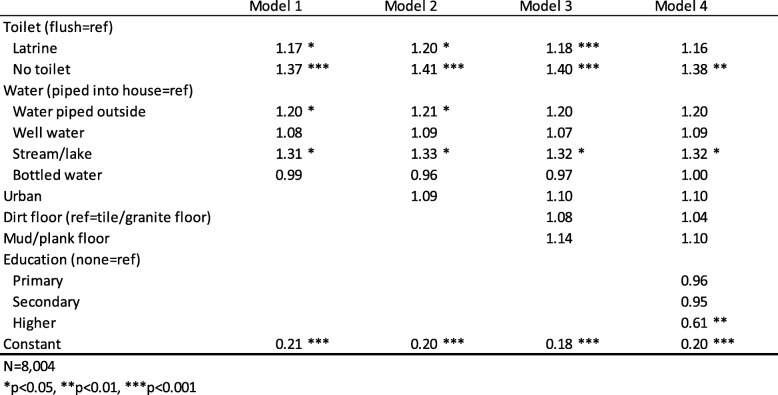
GEE logistic regression odds ratios predicting any child in the household with diarrhea.

**Table 4:**
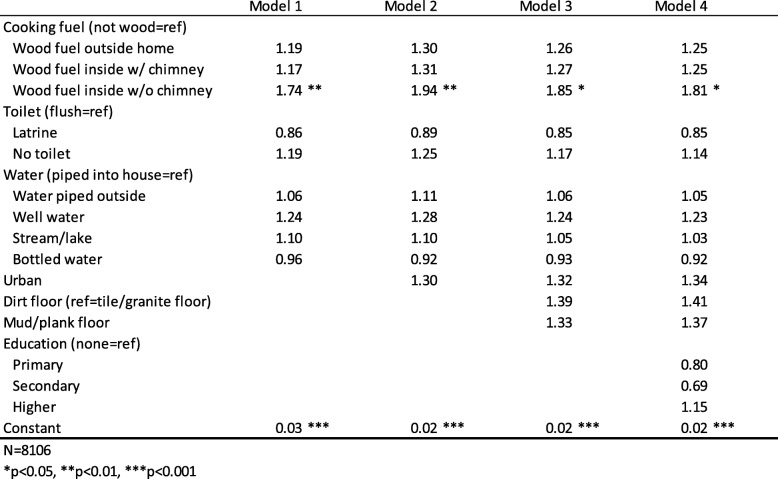
GEE logistic regression odds ratios predicting any infant mortality in the household

As models in Table 2 show, households using wood cooking fuel are 1.2 to 1.7 times more likely to have a child with a cough than those not using wood for fuel. Children living in households without a chimney, where cooking was done using wood inside, have the highest odds of having a cough. This relationship persists in models controlling for urban location, flooring type, and insurance, although flooring type (potentially both a direct contributor to child cough and an indirect indicator of household resources) modestly attenuates the relationship between cooking fuel and child cough. Hypothesis 1 (wood cooking fuel is associated with child cough prevalence) is therefore supported by these analyses.

Table 3 shows the results of generalized equations models predicting households where at least one child under 5 had diarrhea during the previous 2 weeks. Toilet type has a significant and persistent effect throughout the models, with houses with no toilet having 40% higher odds of having a child with diarrhea than those with a flush toilet. These results support Hypothesis 2 that household sanitation affects the risk of child’s diarrhea. Controls are largely not significant in this analysis, although households where the mother’s education is above secondary level are about half as likely to have a child with diarrhea compared to those with no education.

Table 4 shows the results of generalized equations models predicting infant mortality and controlling for household characteristics. This outcome, (infant mortality, see Table 1 for details) is significantly rarer than the other two (child diarrhea and child cough), and this affects the significance of factors in all analyses. Households using wood for fuel indoors, with no chimney, have about 80% higher odds of having an infant death than those not using wood for cooking fuel at all. This result is the only one that supports the expected association in Hypothesis 3, as the rest of the household development measures are not significant. Maternal education and child insurance status are also not significant predictors of infant mortality. Unlike child cough and diarrhea, infant mortality information is collected for children born in the past 5 years (2006–2012) and there is no information regarding where exactly the child was born (in the present household or someplace else). While infant mortality is an important measure of children’s welfare, due to data limitations, the results for this outcome need to be taken cum *grano salis*.

**Fig. 2:**
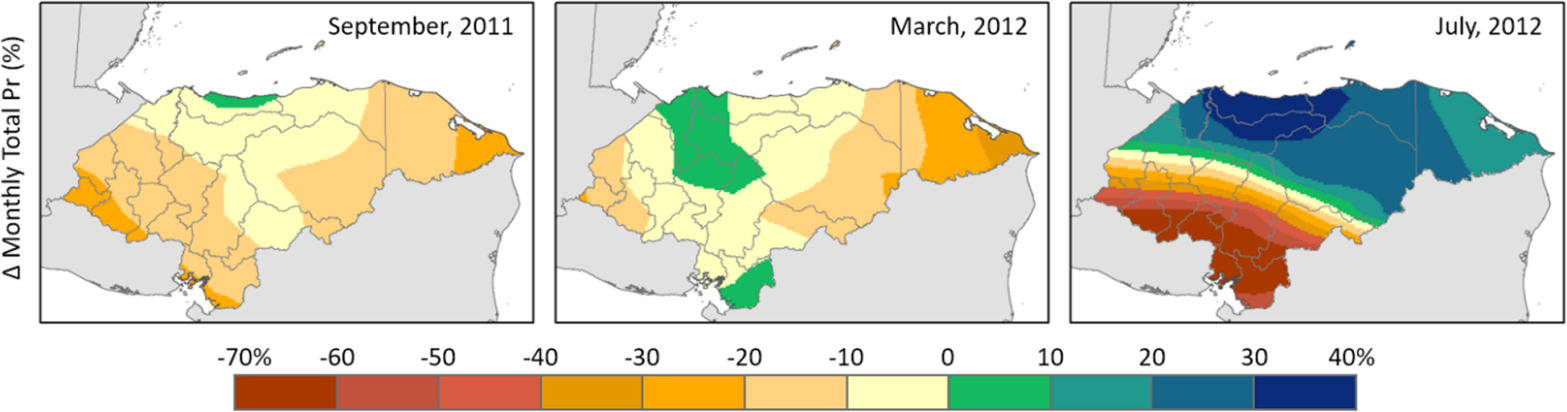
Anomalies (Δ) in monthly total precipitation (Pr; in %) relative to their respective historic (1981–2010) monthly totals. Brown tone indicates drier than average conditions and green-blue tone indicates wetter than average conditions. Three example months are shown here out of the total 10 months of survey data collection period

**Fig. 3:**
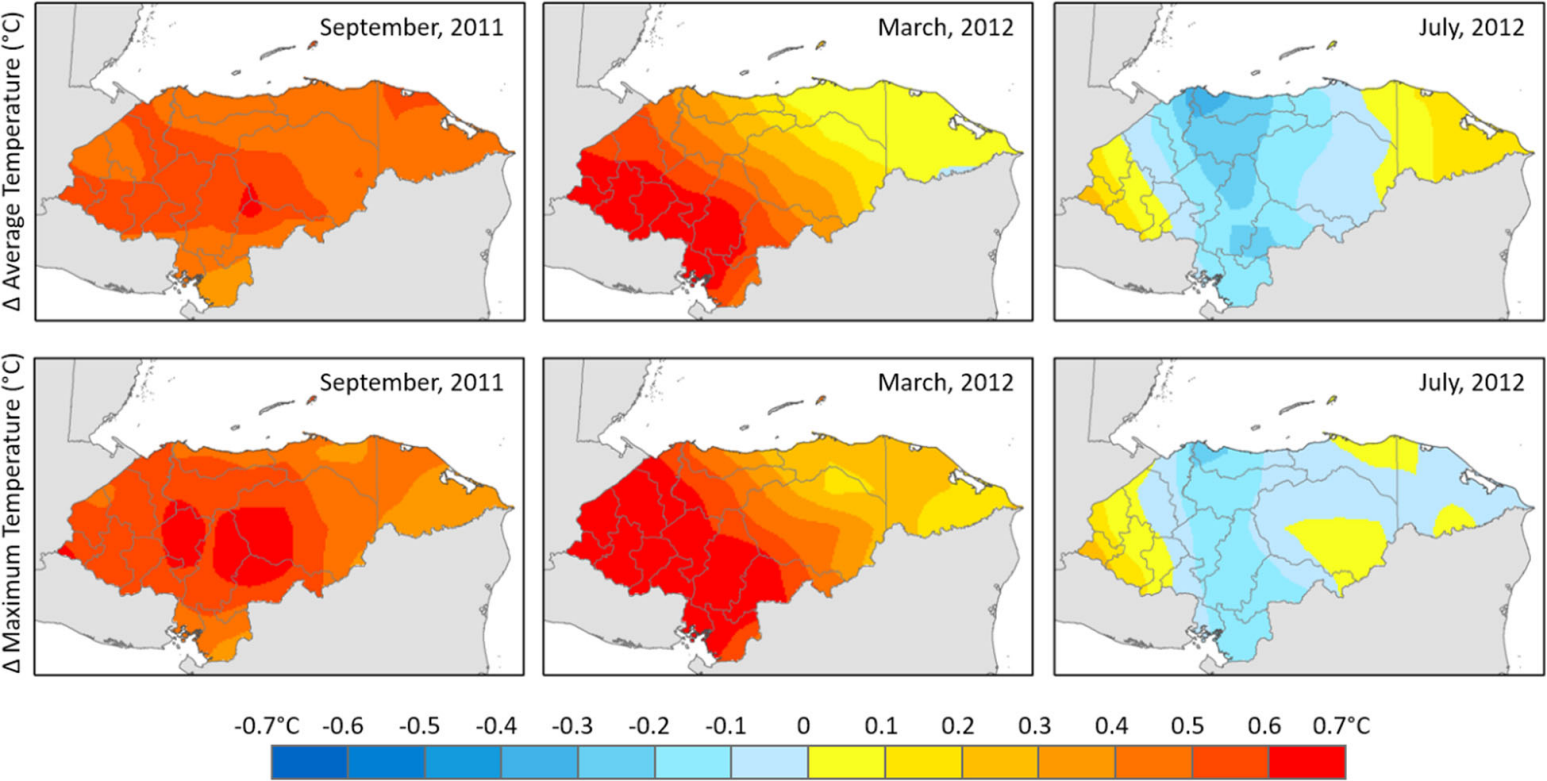
Anomalies (Δ) in monthly temperatures (in °C) relative to their respective historic (1981–2010) monthly temperatures. Yellow-red tone indicates warmer than average temperatures and blue tone indicates cooler than average temperatures. Three example months are shown here out of the total 10 months of survey data collection period

We take into account the limitations for analysis of the type of data that we have and use spatial correlation analysis to link climate variations and child health outcomes. Figures 2 and 3 give a descriptive picture of how temperature and precipitation anomalies are distributed in Honduras.

These figures show that, during the dry season (March–September), the Western part of Honduras tends to become drier and hotter than it has been traditionally.

Table 5 explores the spatial association between climate variables and child health. It shows the values and significance of a correlation coefficient that is modified to take into account that the processes investigated are spatial [41]. We calculated this using SpatialPack in R [42]. In spatial processes, areas that are closer to each other might not be independent and, as such, the sample size might be overestimated. The tests used in here estimate the effective sample size and make adjustments in the variance and degrees of freedom of the t test (used for significance level). The specific levels of the *p* value as well as other statistical measures resulting from this analysis are available in the Additional file 1. This analysis aims to add to our understanding of whether areas experiencing more severe climate anomalies are also those that have higher percentages of children with unfavorable health outcomes.

**Table 5:** Spatial association coefficient, for clusters with 3 or more households

	Percentage of households with			
	child with a cough		child with diarrhea	infant mortality
Precipitation anomaly	0.087	*	0.016	−0.0315
Maximum temperature anomaly	0.0838	*	0.0123	−0.0311
Temperature anomaly	0.0838	*	0.0123	−0.0311
Minimum temperature	0.084	*	0.0123	−0.31
Average temperature	0.0838	*	0.0123	−0.311

Climate related variables (anomalies and mean) are associated with a higher incidence of respiratory illness. Infant mortality and diarrhea incidence do not show any significant association with climate variable. As we have mentioned above, infant mortality information is collected for the past 5 years and it is a much rare outcome than coughing and fever (which affects coefficients’ level of significance). We expected diarrhea to have a significant relation to at least some of the climate measure but it might very well be that this relation is not linear and, therefore, not captured by the methods we have used in this analysis. In sum, the data in Table 5 provide partial support for Hypothesis 4.

## Discussion

In this paper, we have analyzed the linkages between child health, household environment and climate anomalies in order to assess the effects climate change might have on child health in Honduras. We focus on three child health outcomes: cough, diarrhea and infant mortality. Based on literature, we use type of flooring, type of access to clean water and sanitation and type of cooking fuel as measures of household environment. For climate related variables, we examine the relation between the precipitation and temperature (recorded around the area where the household was located) and child health outcomes.

Our analyses show, firstly, that areas experiencing climate anomalies are also the ones with higher incidence of child respiratory problems. Secondly, we identify the household environmental factors that influence the poor child health outcomes – poor sanitation and type of cooking fuel. Tackling in advance those household environmental factors that affect poor child health outcomes (better sanitation and clean cooking fuel) will also function as an adaptation to climate change because climate change induced anomalies in temperature and precipitation will make it more difficult for people to have access to clean water and cooking fuel under current living conditions.

Household environment significantly affects children’s health, child cough and diarrhea incidence, in particular. Use of wood for indoor cooking (in particular, without a chimney) is significantly associated with children’s health outcomes, including cough and infant mortality. By linking climate related data (precipitation and temperature) to child health outcomes, we show that areas with high incidence of child respiratory diseases also experienced higher climate variation in the interval before the interviews took place. Wood fuel, in particular, is a resource vulnerable to climate fluctuations and its shortages may present new health risks for the population such as limiting the means to cook food or boil water and burning other materials that could have an even more toxic effect than wood smoke.

It is fairly well established that climate change will only increase climate variability, so we see Honduras as one of the cases in which co-benefits actions (access to clean energy and improved sanitation) would not only have a significant effect on children’s health but would also function as an adaptation to climate change [43–45]. However, this needs to be done in fairly systematic way either by the Honduran government or through international aid agencies.

If there are no interventions of any kind, we envision two possible paths for the health outcomes analyzed. The first one would be an accelerated rate of economic growth (industrialization, intensive agriculture, and increasing urbanization) that would significantly increase the wealth of even the poorest individuals in Honduras. While such a level and type of development is highly unlikely in Honduras, the health risks at the household level will indeed decrease (fewer people will have bad sanitation, for example). At the same time, the health risks brought by urbanization and increasing industrialization will follow an inverse U shape (increase and then decrease) while the global effects (increasing greenhouse emissions) will worsen the effects of climate change [5].

In the second scenario (household wealth doesn’t increase significantly in Honduras), migration might be the main options taken by people whose livelihoods and health are affected by climate change. As studies have shown [8, 21, 46], significant migratory movements have already occurred in Honduras (internally- rural to urban- as well as internationally) and climate change has an influence on the decision to migrate [8]. Climate changes, as well as social factors (such as violence), are likely to push more individuals either toward the major urban centers of Honduras or internationally. We anticipate that, under this scenario, the poorest families left in Honduras will still be living in unhealthy conditions. That is, the percentage of families with access to clean water and with adequate wastewater sanitation is likely to decrease, whereas the aforementioned potential depletion of firewood for cooking may reduce ability to safely cook food. Rural to urban migrants (as well as international migrants) tend to rely on social networks to migrate and if some families move into unplanned settlements, then those who follow them would be more likely to also settle next to them ([21]; Lomnitz, 1977).

## Conclusions

This research shows that climate anomalies have a significant effect on children’s respiratory issues in Honduras. As climate change will only enhance the incidence of climate anomalies, in the absence of improved socio-economic conditions, these issues will only become worse.

## Supplementary information

**Additional file 1.**

## Data Availability

All the data used in here are publicly available.

## Footnotes

1 https://climatedataguide.ucar.edu/climate-data/cru-ts321-gridded-precipitation-and-other-meteorological-variables-1901
