# Supplementary material for: Child health, household environment, temperature and rainfall anomalies in Honduras: a socio-climate data linked analysis
Source: Environ Health. 2020 Jan 28;19:10. doi: 10.1186/s12940-020-0560-9 (PMC6986158; doi:10.1186/s12940-020-0560-9)
Supplement: Supplementary file 1 — Additional file 1. [file 12940_2020_560_MOESM1_ESM.docx]

**Appendix**

Supplementary information, corrected Pearson's correlation for spatial autocorrelation calculated using SpatialPack in R (Table 5). Nclass for Moran I set at 13 for all sets of variables.

1. *Coughing – precipitation anomaly*

F-statistic: 5.6145 on 1 and 723.4273 DF, p-value: 0.0181

**Class Upper Cardinality Moran index**

**Bounds x y**

1 0.4695 56213 0.0253925 0.246503

2 0.9391 109822 -0.0192460 0.012707

3 1.4086 128903 -0.0048912 0.013448

4 1.8781 131484 0.0137856 -0.020742

5 2.3477 96968 -0.0094476 -0.034444

6 2.8172 52267 0.0004084 -0.092162

7 3.2867 24222 -0.0004725 -0.232995

8 3.7563 10470 -0.0177574 -0.065662

9 4.2258 6378 -0.0071619 -0.035198

10 4.6953 4887 0.0191059 -0.006382

11 5.1649 2309 0.0011196 -0.030869

12 5.6344 1318 -0.0147251 -0.006899

13 6.1039 280 0.0210988 0.021978

1. *Coughing – maximum temperature anomaly*

F-statistic: 5.0432 on 1 and 712.4474 DF, p-value: 0.025

**Class Upper Cardinality Moran index**

**Bounds x y**

1 0.4695 56213 0.0253925 0.246845

2 0.9391 109822 -0.0192460 0.008458

3 1.4086 128903 -0.0048912 0.012114

4 1.8781 131484 0.0137856 -0.018585

5 2.3477 96968 -0.0094476 -0.034304

6 2.8172 52267 0.0004084 -0.087765

7 3.2867 24222 -0.0004725 -0.231920

8 3.7563 10470 -0.0177574 -0.063253

9 4.2258 6378 -0.0071619 -0.032288

10 4.6953 4887 0.0191059 -0.003373

11 5.1649 2309 0.0011196 -0.028925

12 5.6344 1318 -0.0147251 -0.004754

13 6.1039 280 0.0210988 0.024678

1. *Coughing –temperature anomaly*

F-statistic: 5.0422 on 1 and 712.4417 DF, p-value: 0.025

**Class Upper Cardinality Moran index**

**Bounds x y**

1 0.4695 56213 0.0253925 0.246843

2 0.9391 109822 -0.0192460 0.008444

3 1.4086 128903 -0.0048912 0.012113

4 1.8781 131484 0.0137856 -0.018589

5 2.3477 96968 -0.0094476 -0.034285

6 2.8172 52267 0.0004084 -0.087759

7 3.2867 24222 -0.0004725 -0.231924

8 3.7563 10470 -0.0177574 -0.063267

9 4.2258 6378 -0.0071619 -0.032279

10 4.6953 4887 0.0191059 -0.003341

11 5.1649 2309 0.0011196 -0.028915

12 5.6344 1318 -0.0147251 -0.004743

13 6.1039 280 0.0210988 0.024718

1. *Coughing –minimum temperature*

F-statistic: 5.0386 on 1 and 712.5186 DF, p-value: 0.0251

**Class Upper Cardinality Moran index**

**Bounds x y**

1 0.4695 56213 0.0253925 0.246804

2 0.9391 109822 -0.0192460 0.008242

3 1.4086 128903 -0.0048912 0.011990

4 1.8781 131484 0.0137856 -0.018780

5 2.3477 96968 -0.0094476 -0.034001

6 2.8172 52267 0.0004084 -0.087094

7 3.2867 24222 -0.0004725 -0.232205

8 3.7563 10470 -0.0177574 -0.063176

9 4.2258 6378 -0.0071619 -0.031863

10 4.6953 4887 0.0191059 -0.002612

11 5.1649 2309 0.0011196 -0.028171

12 5.6344 1318 -0.0147251 -0.004145

13 6.1039 280 0.0210988 0.025343

1. *Coughing –average temperature*

F-statistic: 5.0361 on 1 and 712.5196 DF, p-value: 0.0251

**Class Upper Cardinality Moran index**

**Bounds x y**

1 0.4695 56213 0.0253925 0.246821

2 0.9391 109822 -0.0192460 0.008317

3 1.4086 128903 -0.0048912 0.012027

4 1.8781 131484 0.0137856 -0.018709

5 2.3477 96968 -0.0094476 -0.034018

6 2.8172 52267 0.0004084 -0.087184

7 3.2867 24222 -0.0004725 -0.232692

8 3.7563 10470 -0.0177574 -0.063372

9 4.2258 6378 -0.0071619 -0.032072

10 4.6953 4887 0.0191059 -0.002791

11 5.1649 2309 0.0011196 -0.028400

12 5.6344 1318 -0.0147251 -0.004335

13 6.1039 280 0.0210988 0.025145

1. *Child with diarrhea –precipitation anomaly*

F-statistic: 0.1023 on 1 and 763.4039 DF, p-value: 0.7491

**Class Upper Cardinality Moran index**

**Bounds x y**

1 0.4695 56213 0.018229 0.246503

2 0.9391 109822 0.010195 0.012707

3 1.4086 128903 -0.007118 0.013448

4 1.8781 131484 -0.012610 -0.020742

5 2.3477 96968 0.003764 -0.034444

6 2.8172 52267 -0.002214 -0.092162

7 3.2867 24222 0.003238 -0.232995

8 3.7563 10470 -0.047718 -0.065662

9 4.2258 6378 -0.045766 -0.035198

10 4.6953 4887 -0.016576 -0.006382

11 5.1649 2309 0.106843 -0.030869

1. 5.6344 1318 0.116083 -0.006899

13 6.1039 280 0.060135 0.021978

1. *Child with diarrhea –maximum temperature anomaly*

F-statistic: 0.116 on 1 and 770.4412 DF, p-value: 0.7335

**Class Upper Cardinality Moran index**

**Bounds x y**

1 0.4695 56213 0.018229 0.246845

2 0.9391 109822 0.010195 0.008458

3 1.4086 128903 -0.007118 0.012114

4 1.8781 131484 -0.012610 -0.018585

5 2.3477 96968 0.003764 -0.034304

6 2.8172 52267 -0.002214 -0.087765

7 3.2867 24222 0.003238 -0.231920

8 3.7563 10470 -0.047718 -0.063253

9 4.2258 6378 -0.045766 -0.032288

10 4.6953 4887 -0.016576 -0.003373

11 5.1649 2309 0.106843 -0.028925

1. 5.6344 1318 0.116083 -0.004754

13 6.1039 280 0.060135 0.024678

1. *Child with diarrhea –temperature anomaly*

F-statistic: 0.116 on 1 and 770.4412 DF, p-value: 0.7336

sample correlation: 0.0123

**Class Upper Cardinality Moran index**

**Bounds x y**

1 0.4695 56213 0.018229 0.246844

2 0.9391 109822 0.010195 0.008450

3 1.4086 128903 -0.007118 0.012114

4 1.8781 131484 -0.012610 -0.018587

5 2.3477 96968 0.003764 -0.034295

6 2.8172 52267 -0.002214 -0.087762

7 3.2867 24222 0.003238 -0.231920

8 3.7563 10470 -0.047718 -0.063261

9 4.2258 6378 -0.045766 -0.032282

10 4.6953 4887 -0.016576 -0.003356

11 5.1649 2309 0.106843 -0.028919

12 5.6344 1318 0.116083 -0.004748

13 6.1039 280 0.060135 0.024701

1. *Child with diarrhea –minimum temperature*

F-statistic: 0.117 on 1 and 770.1834 DF, p-value: 0.7325

**Class Upper Cardinality Moran index**

**Bounds x y**

1 0.4695 56213 0.018229 0.246804

2 0.9391 109822 0.010195 0.008242

3 1.4086 128903 -0.007118 0.011990

4 1.8781 131484 -0.012610 -0.018780

5 2.3477 96968 0.003764 -0.034001

6 2.8172 52267 -0.002214 -0.087094

7 3.2867 24222 0.003238 -0.232205

8 3.7563 10470 -0.047718 -0.063176

9 4.2258 6378 -0.045766 -0.031863

10 4.6953 4887 -0.016576 -0.002612

11 5.1649 2309 0.106843 -0.028171

12 5.6344 1318 0.116083 -0.004145

1. 6.1039 280 0.060135 0.025343
2. *Child with diarrhea –average temperature*

F-statistic: 0.1162 on 1 and 770.2422 DF, p-value: 0.7333

**Class Upper Cardinality Moran index**

**Bounds x y**

1 0.4695 56213 0.018229 0.246821

2 0.9391 109822 0.010195 0.008317

3 1.4086 128903 -0.007118 0.012027

4 1.8781 131484 -0.012610 -0.018709

5 2.3477 96968 0.003764 -0.034018

6 2.8172 52267 -0.002214 -0.087184

7 3.2867 24222 0.003238 -0.232692

8 3.7563 10470 -0.047718 -0.063372

9 4.2258 6378 -0.045766 -0.032072

10 4.6953 4887 -0.016576 -0.002791

11 5.1649 2309 0.106843 -0.028400

12 5.6344 1318 0.116083 -0.004335

13 6.1039 280 0.060135 0.025145

1. *Infant mortality –precipitation anomaly*

F-statistic: 1.0567 on 1 and 1063.098 DF, p-value: 0.3042

**Class Upper Cardinality Moran index**

**Bounds x y**

1 0.4695 56213 0.0002911 0.246503

2 0.9391 109822 -0.0016053 0.012707

3 1.4086 128903 -0.0002899 0.013448

4 1.8781 131484 -0.0019114 -0.020742

5 2.3477 96968 0.0006777 -0.034444

6 2.8172 52267 0.0049797 -0.092162

7 3.2867 24222 -0.0089793 -0.232995

8 3.7563 10470 -0.0248395 -0.065662

9 4.2258 6378 0.0065542 -0.035198

10 4.6953 4887 -0.0153899 -0.006382

11 5.1649 2309 0.0454249 -0.030869

12 5.6344 1318 -0.0172764 -0.006899

13 6.1039 280 -0.0286308 0.021978

1. *Infant mortality –* *Maximum temperature anomaly*

F-statistic: 1.0274 on 1 and 1061.48 DF, p-value: 0.311

**Class Upper Cardinality Moran index**

**Bounds x y**

1 0.4695 56213 0.0002911 0.246845

2 0.9391 109822 -0.0016053 0.008458

3 1.4086 128903 -0.0002899 0.012114

4 1.8781 131484 -0.0019114 -0.018585

5 2.3477 96968 0.0006777 -0.034304

6 2.8172 52267 0.0049797 -0.087765

7 3.2867 24222 -0.0089793 -0.231920

8 3.7563 10470 -0.0248395 -0.063253

9 4.2258 6378 0.0065542 -0.032288

10 4.6953 4887 -0.0153899 -0.003373

11 5.1649 2309 0.0454249 -0.028925

12 5.6344 1318 -0.0172764 -0.004754

13 6.1039 280 -0.0286308 0.024678

1. *Infant mortality –* *Temperature anomaly*

F-statistic: 1.0267 on 1 and 1061.463 DF, p-value: 0.3112

**Class Upper Cardinality Moran index**

**Bounds x y**

1 0.4695 56213 0.0002911 0.246843

2 0.9391 109822 -0.0016053 0.008444

3 1.4086 128903 -0.0002899 0.012113

4 1.8781 131484 -0.0019114 -0.018589

5 2.3477 96968 0.0006777 -0.034285

6 2.8172 52267 0.0049797 -0.087759

7 3.2867 24222 -0.0089793 -0.231924

8 3.7563 10470 -0.0248395 -0.063267

9 4.2258 6378 0.0065542 -0.032279

10 4.6953 4887 -0.0153899 -0.003341

11 5.1649 2309 0.0454249 -0.028915

12 5.6344 1318 -0.0172764 -0.004743

13 6.1039 280 -0.0286308 0.024718

1. *Infant mortality –minimum temperature*

F-statistic: 1.0233 on 1 and 1060.818 DF, p-value: 0.312

**Class Upper Cardinality Moran index**

**Bounds x y**

1 0.4695 56213 0.0002911 0.246804

2 0.9391 109822 -0.0016053 0.008242

3 1.4086 128903 -0.0002899 0.011990

4 1.8781 131484 -0.0019114 -0.018780

5 2.3477 96968 0.0006777 -0.034001

6 2.8172 52267 0.0049797 -0.087094

7 3.2867 24222 -0.0089793 -0.232205

8 3.7563 10470 -0.0248395 -0.063176

9 4.2258 6378 0.0065542 -0.031863

10 4.6953 4887 -0.0153899 -0.002612

11 5.1649 2309 0.0454249 -0.028171

12 5.6344 1318 -0.0172764 -0.004145

13 6.1039 280 -0.0286308 0.025343

1. *Infant mortality –average temperature*

F-statistic: 1.0248 on 1 and 1060.727 DF, p-value: 0.3116

**Class Upper Cardinality Moran index**

**Bounds x y**

1 0.4695 56213 0.0002911 0.246821

2 0.9391 109822 -0.0016053 0.008317

3 1.4086 128903 -0.0002899 0.012027

4 1.8781 131484 -0.0019114 -0.018709

5 2.3477 96968 0.0006777 -0.034018

6 2.8172 52267 0.0049797 -0.087184

7 3.2867 24222 -0.0089793 -0.232692

8 3.7563 10470 -0.0248395 -0.063372

9 4.2258 6378 0.0065542 -0.032072

10 4.6953 4887 -0.0153899 -0.002791

11 5.1649 2309 0.0454249 -0.028400

12 5.6344 1318 -0.0172764 -0.004335

13 6.1039 280 -0.0286308 0.025145
